# Top of the license practice or out of our scope? A qualitative analysis of social workers’ role in cultivating prognostic awareness on hospital palliative care teams

**DOI:** 10.1186/s12904-026-02028-w

**Published:** 2026-02-25

**Authors:** Arden E. O’Donnell, Taylor B. Wensley, Judith G. Gonyea

**Affiliations:** 1 Boston University School for Social Work, Boston, MA United States; 2 MassGeneralBrigham Hospital, MA Boston, United States

**Keywords:** prognostic awareness, goals of care, palliative care, serious illness, end of life, palliative social work, interprofessional collaboration, role theory, qualitative research

## Abstract

**Objective:**

Prognostic awareness, defined as patients’ understanding of their illness trajectory and likely prognosis, is an essential component of informed decision-making, values-based care planning, and high-quality palliative care. Although discussing prognosis is traditionally viewed as a physician-led task, palliative social workers (PSWs) are often involved in interventions that require prognostic understanding. Little is known, however, about the role social workers play in cultivating prognostic awareness, the professional boundaries surrounding prognostic discussions, and how PSWs’ engagement in this work is understood within interprofessional palliative care teams.

**Methods:**

This qualitative study used Interpretive Description, an applied analytic approach designed to generate practice-relevant understanding grounded in participants’ experiential accounts. Semi-structured interviews were conducted with 17 advanced-practice (APHSW-certified) palliative social workers and 16 palliative physicians practicing in U.S. hospital settings. Inductive thematic analytic techniques were used to identify patterned understandings across participants’ accounts. Role theory was used as a sensitizing framework to support interpretation of interprofessional expectations, boundaries, and dynamics related to cultivating prognostic awareness.

**Results:**

Across professions, participants described cultivating prognostic awareness as an iterative, team-based process within inpatient palliative care. PSWs were described as extending and deepening physician-initiated conversations by reinforcing prognostic information, exploring lived experience, and supporting emotional processing. Clear professional boundaries were articulated around prognostic formulation, initial time-based disclosure, and medically complex decision-making, which were widely viewed as physician led. Role tensions emerged when workflow pressures, clinical urgency, or team expectations pushed PSWs toward medically focused conversations, or when primary teams assumed PSWs could independently deliver prognostic information. Interprofessional trust, communication, and team alignment shaped whether PSWs were supported to engage fully in this work.

**Conclusions:**

Engagement in cultivating prognostic awareness is widely viewed as top-of-license practice for specialty-trained PSWs when grounded in their psychosocial expertise, communication skills, and collaboration with physicians. Role agreement, organizational support, and interprofessional trust are essential for enabling PSWs to practice within their scope while maximizing their unique contributions to patient and family prognostic understanding.

**Supplementary Information:**

The online version contains supplementary material available at 10.1186/s12904-026-02028-w.

## Introduction

The landscape of hospital-based palliative care has grown rapidly in recent years, yet persistent gaps remain between patient needs and available resources [1–3]. These pressures reinforce the mandate for clinicians to practice at the top of their license to provide high-quality, patient-centered care [4–6]. As palliative care programs expand, many are increasingly relying on palliative social workers (PSWs), particularly those who hold advanced credentials such as the Advanced Palliative and Hospice Social Work certification (APHSW-C). This certification signals specialist-level practice through rigorous requirements, including a post-graduate degree in social work, clinical licensure, over 4,000 h of supervised hospice and/or palliative care practice within the past five years, before successful completion of an evidence-based certification examination [7–9]. This professionalization reflects a growing distinction between generalist and specialist-level skills and calls for renewed attention to the boundaries and expectations that define specialty palliative practice [5, 10, 11]. This is especially important in hospital-based palliative care, where interprofessional collaboration often requires role-sharing [12–14].

One area of expanding scope involves PSWs’ participation in cultivating prognostic awareness, a process by which patients and families come to understand the likely trajectory of illness and its prognosis [15]. Prognostic awareness is crucial because it fosters more timely decision making, improves quality of life, and helps align care with patients’ values and goals [16–18]. An accurate prognostic understanding is not only important for disease-based treatment decisions but also integral to many PSWs’ core interventions, including advance care planning, goals-of-care conversations, legacy work, and referrals to hospice [5, 11]. Historically, prognostic understanding was viewed as the outcome of a single, physician-led disclosure focused on medical facts and timelines. Increasingly, however, prognostic understanding is recognized as an iterative process grounded in skilled communication, emotional attunement, and compassion [15, 19–21]. As such, cultivating prognostic awareness may be a shared responsibility across professions [22–25], particularly in acute care settings where interprofessional palliative care (IPC) teams are often consulted to support decision making in the context of acute changes in illness and/or disease progression.

As PSWs’ scope of practice expands, engagement in prognostic discussions is likely to become an area of interprofessional role negotiation, as expectations, boundaries, and responsibilities play out in real-world clinical settings. Conversations involving prognosis are particularly vulnerable to role tension because they sit at the intersection of medical authority and psychosocial meaning-making. While physicians are traditionally responsible for prognostic disclosures, cultivating prognostic awareness requires exploration of patients and families understanding of illness trajectory, their values, emotions, and current and past lived experiences, all areas central to PSW’s clinical practice. This overlap can create ambiguity around professional boundaries and expectations, making engagement in the process of cultivating prognostic awareness a key area of role sharing as well as potential role conflict within IPC teams.

Existing research underscores PSWs’ important contributions to goals-of-care discussions [26–29], advance care planning [30, 31], family meetings [8, 12], and discharge planning [28]. However, most studies focus on patient outcomes and do not explicitly examine PSWs’ involvement or role in discussions surrounding prognosis. In addition, the limited literature examining how IPC teams collectively discuss and negotiate prognosis focuses primarily on physicians and nurses [23, 32–36]. For example, a recent scoping review of prognostic decision-making within multidisciplinary teams found that only 4 of 40 included studies examined social workers involvement, highlighting a gap in the literature [33].

This study seeks to broaden this conversation by examining how PSWs and physicians understand the boundaries, shared responsibilities, and professional expectations related to PSWs’ engagement in prognostic conversations. Specifically, this research study asks: Is cultivating prognostic awareness viewed as part of specialist PSWs’ clinical practice, and, if so, what team, organizational, and professional dynamics emerge if PSW’s engage in this practice? Addressing these questions may inform education and help clarify interprofessional role expectations and support practice that reflects the full scope of PSW’s expertise.

### Study design

This study used Interpretive Description (ID) [37, 38], a qualitative approach developed for applied health disciplines to generate practice‑relevant understanding grounded in participants’ experiential accounts. ID has been widely used in nursing, palliative care, and medical education and is well‑suited to examining complex practice‑based phenomena such as interprofessional role negotiation [39–41]. To examine how the conceptualization of prognostic awareness as a team‑based process may shape collaborative practice within hospital‑based interprofessional palliative care (IPC) teams, we used Role Theory [42, 43] as a sensitizing framework, attending to role agreement, valuing, sharing, and ambiguity within IPC teams.

### Recruitment, sampling, and data collection

Participants were recruited through a two-step process. First, hospital-based PSWs were recruited via email from a national registry of 750 APHSW-certified social workers. Second, following their interview, PSWs identified up to two palliative care team members (including at least one physician) who had worked closely with them in the prior six months. After informed consent, semi-structured interviews (see Supplementary Materials) were conducted via Zoom, audio-recorded, transcribed verbatim, and de-identified. Interviews averaged approximately 60 min for PSWs and 20 min for physicians, reflecting differences in role focus and clinical availability. Participants received a $25 gift card.

### Analytic approach

Analysis followed an Interpretive Description (ID) methodology [37, 38], using iterative, inductive coding and thematic patterning to develop an interpretive account of how PSWs and physicians perceived, understood, and navigated PSWs’ engagement in cultivating prognostic awareness. First, all authors engaged in repeated readings of transcripts to deepen immersion in participants’ accounts. AO and TW then conducted inductive line‑by‑line coding across the full dataset in NVivo, using codes to organize and explore patterns within and across interviews rather than to confirm reliability. Codes were iteratively reviewed, compared, and clustered into groupings that captured shared and divergent aspects of participants’ accounts within and across professional groups. A third team member (JG), a non‑clinician health researcher, reviewed a subset of transcripts to confirm and expand the interpretive lens. Through iterative team dialogue, these patterns were refined into broader interpretive themes that moved beyond description to consider practice‑relevant understandings of how prognostic awareness work was conceptualized, enacted, constrained, and negotiated during palliative care consults.

In a subsequent interpretive phase, Biddle’s role theory [42, 43] was used as a synthesizing framework to deepen the understanding of the inductively derived themes. We drew on role‑theory constructs of role agreement, role ambiguity, role tension/conflict, role valuing, and role sharing to consider interprofessional dynamics rather than to dictate thematic structure. We understood *role agreement* as the extent to which expectations, responsibilities, and boundaries around PSWs’ prognostic work were clearly articulated, and *role ambiguity* as uncertainty or inconsistency in those expectations. *Role conflict/tension* described situations in which PSWs faced incompatible or competing expectations (for example, being asked both to avoid and to lead prognostic conversations), producing tension and strain. We used *role valuing* to capture the degree to which PSWs’ roles were recognized, respected, and seen as legitimate within teams, and *role sharing* to describe overlapping or jointly enacted responsibilities for cultivating prognostic awareness across professions.

Authors these constructs to sharpen interpretation and organize the narrative. For example, we examined where participants described consensus that cultivating prognostic awareness as being within PSWs’ role (role clarity), where they described explicit limits around that engagement (role boundaries), how responsibilities were distributed or shared between PSWs and physicians (role sharing), and where tensions in expectations suggested the potential for role conflict. Accounts that did not align neatly with Role Theory constructs were retained and used to refine theme boundaries and to highlight ambiguity, tension, and complexity in role expectations, rather than being excluded. Ongoing reflexive dialogue within the research team supported theme development, including revising, combining, and re‑working themes as analysis progressed. Consistent with Interpretive Description, we conceptualized quality in terms of coherence, interpretive depth, and relevance to clinical practice rather than validation or generalizability. Analytic rigor was supported through regular team discussions, documentation of analytic decisions, and reflexive attention to researcher positionality. Specifically, AO’s position as a practicing PSW was treated as an analytic resource and explicitly examined through reflexive journaling and regular discussion with her academic and clinical mentor (a palliative physician).

### Ethics

This study received institutional review board approval from Boston University and was conducted in accordance with the ethical principles of the Declaration of Helsinki and applicable institutional guidelines. Participation was voluntary, and all participants provided informed consent prior to participation.

### Findings

#### Study sample

Table 1 provides the participants’ demographic information. Participants were predominantly white (76%) and female (73%). Although from diverse geographic regions of the U.S., 76% worked at urban teaching hospitals. All interviewees were experienced in palliative care with over 80% of physicians having worked in palliative care for five or more years, and 100% of PSW’s working for more than 5 years, with 40% having worked for more than a decade.

**Table 1:** Demographics

Baseline Characteristic	*N* = 33	%
Profession		
Social workers	17	52%
Physician	16	48%
Gender		
Female	24	73%
Male	9	27%
Race		
White	25	76%
Black	1	3%
Latinx	2	6%
Asian/Southeast Asian	5	15%
Years in Palliative Care		
1–4 years (all MD’s)	6	18%
5–10 years	16	48%
11 + years	11	34%
Hospital Setting		
Community Hospital	8	24%
Urban Teaching Hospital	25	76%
Geographic Area		
New England	9	27%
Midwest	7	21%
South/Southeast	11	33%
West Coast	6	18%

### Findings

Findings are organized using role theory as an interpretive lens. Section headings highlight practice-based patterns in participants’accounts, with role theory constructs noted parenthetically to support interpretation.

### Negotiating PSWs’ role in prognostic awareness (role clarity and agreement)

#### Cultivating prognostic awareness is within PSWs’ scope of practice

Participants’ accounts highlighted how palliative social workers’ involvement in cultivating prognostic awareness was generally understood as part of advanced palliative practice, while also revealing variation in how clearly this role was articulated and supported across clinical contexts. Physicians described it as *“an essential part of what we do as a palliative care team*,*”* emphasizing that “*it takes everyone*,* especially the social worker*,* to help patients and families make sense of what’s ahead”* (MD 10). PSWs similarly characterized this work as requiring experience and training, with one noting,

*“Cultivating prognostic awareness is an advanced palliative care skill. I wouldn’t expect somebody on the first day or even the first year on the job to feel comfortable exploring at the level that I am after almost 10 years”* (PSW 2).

PSWs framed prognostic awareness as inseparable from goals-of-care work. As one PSW explained, *“prognostic awareness is intimately tied to what their goals may or may not be*,* you can’t tease it out and leave it separate*.*”* positioning patients’ understanding of illness trajectory as foundational to values clarification and decision making. Physicians described a parallel shift in how prognostication is conceptualized within palliative care, moving beyond a singular focus on time toward functional prognosis and uncertainty as forms of prognostic disclosure. One physician described this as *“help[ing] paint a picture for what the future could look like*,* continue to revise it*,* cope as best we can*,* and move it along*,*”* rather than simply conveying *“how much time they have left*,*”* noting that this approach *“is different and takes a high level of skill”* (MD 16). Of note, a small number of interviewed PSWs working in community hospitals without full time physician coverage expressed uncertainty about whether cultivating prognostic awareness would be supported as part of their role, highlighting that role clarity was shaped not only by professional scope but also by organizational context.

### Drawing boundaries around prognostic work (role boundaries)

#### Time-based prognosis and medically complex information

Participants uniformly described clear professional role boundaries regarding prognostic disclosure. Physicians were viewed as responsible for determining and communicating time-based prognosis and medically complex information, and PSWs consistently emphasized avoiding independent engagement in medical discussions. One physician noted, *“I would be concerned if the social worker gave a strong opinion on a medical procedure or talked about time without physician input”* (MD 4). PSWs similarly articulated these limits, emphasizing that prognostic formulation fell outside their scope, while interpretation and support did not. As one PSW explained,

*“I’m not looking at a medical chart and coming up with a prognosis on my own—that is out of our scope… but if the doctor is telling me what the prognosis is*,* it is 100% within my scope of practice to take that information and translate it into my work with a family”* (PSW 2).

### Navigating consultative boundaries with primary teams

Both professions emphasized the importance of respecting the boundaries linked to palliative care’s consultative role and the importance of maintaining alignment with primary treating teams. Participants described navigating boundaries when primary teams limited prognostic discussion required careful judgment about when to defer and when patient understanding might be compromised if they did not speak out. As one PSW reflected, *“there are times when the primary team asks us not to get into certain details about prognosis… and we have to decide if we will respect their request or are we doing a disservice to the patient if we withhold too much”* (PSW 15).

### Shared and collaborative prognostic work across professions (role sharing)

#### Joint consults as collaborative encounters

Both PSWs and physicians described joint patient and/or family consultations as a primary site of role sharing, requiring fluid coordination and mutual responsiveness. One PSW characterized these encounters as *“a dance… it’s collaborative*,*”* noting that *“sometimes I ask a doctor-type question*,* and they ask one more psychosocial”* (PSW 2). Physicians echoed this framing, describing prognostic communication as *“a team sport*,*”* acknowledging that when an individual delivers all information, it can become *“an emotional avalanche*,*”* with social workers helping to support both the patient and the family (MD 17).

#### Extending and deepening physicians’ conversations

PSWs were also described as playing a key role in extending prognostic conversations beyond formal visits. One PSW explained, *“I often circle back later… helping them process and move through difficult emotions so they can hear and understand the prognostic information”* (PSW 8). Physicians valued this contribution, with one noting that social workers often have *“deeper and more difficult conversations than we even have in our meetings”* which help families process emotionally difficult information (MD 5a).

#### Centering lived experience and functional prognosis discussions

PSWs described exploring and centering patients’ lived experience and exploring functional changes rather than time. As one PSW explained, *“I explore their lived experience*,* then give a functional prognosis… ‘you’ve been in the hospital three times in the last four months*,* I’m worried this is the best it’s going to get’ which sort of says time is getting shorter*,* but not directly”* (PSW 15). Physicians noted that this approach was often received differently by patients, observing that when social workers discuss *“functional decline or what support might be needed*,*”* it is *“seen differently”* and “*feels less threatening”* than when similar information comes from physicians (MD 3a).

#### Navigating tensions and competing expectations in practice (role tension and potential role conflict)

Participants’ accounts also reflected situations in which expectations surrounding prognostic conversations became misaligned, creating tensions that required PSWs to navigate competing demands, shift responsibilities, and face ethical concerns in real-time clinical practice.

#### Primary team expectations and shifting role demands

Physicians and PSWs described situations in which expectations from consulting teams extended beyond what participants understood as traditional social work scope. Physicians expressed concern when PSWs were expected to lead medical conversations about prognosis, such as code status discussions. One physician reflecting on this role tension, posited *“But they are seen as palliative care*,* so maybe it is expected?”* (MD 15) PSWs similarly noted that their identity as palliative care clinicians shaped expectations from both medical colleagues and other social workers. Several described being expected to have a deeper understanding of medical information because of their specialty role, stating *“Understanding clinical progression of disease is expected and valued… even other social workers come to me asking about medical procedures and options.”* (PSW 4) In some cases, PSWs’ professional identity as social workers was less visible to other clinicians. Two non–palliative care physicians noted that they initially *“did not even know she was a social worker”* suggesting that PSWs were sometimes perceived primarily as a palliative care clinician rather than a social worker.

#### Workflow pressures and clinical urgency

Workflow pressures and a desire to support families during crisis situations(e.g., during active coding, acute deterioration, initiation of ECMO) often drove role PSWs to step outside their traditional role boundaries. PSWs described adapting their role to support the team as well as meeting the needs of families when teams were *“swamped*,*”* explaining that they might *“lay the groundwork… then circle back later together to have a deeper conversation”* (PSW 9) or attend family meetings as the sole palliative care provider.

#### Working with less experienced clinicians

Patterns of potential role conflict were especially evident when PSWs worked with learners or less experienced clinicians. Physicians described observing PSWs redirecting or guiding conversations toward prognosis when working with learners, with one noting that social workers would suggest, *“maybe it would be helpful if you talk a little about how this is going to look in the future*,*”* describing this approach as *“really effective*,* particularly with less experienced providers”* (MD 16). PSWs similarly described coaching clinicians to use clear language, noting that *“when I’m with newer doctors*,* I will coach them ‘Say the word death*,*’ or I’ll even write it and slide it to the doctor… but sometimes you just have to say it directly*” (PSW 13).

#### Ethical obligations and patient advocacy

PSWs described ethical tensions when prognostic information was avoided or withheld, citing professional commitments to truth-telling and patient advocacy. One PSW explained, *“I had to say something… it was unethical to let her keep asking without an answer”* (PSW 11). Another reflected on situations in which teams avoided acknowledging dying, stating that these moments “*drive me to say what no one else is saying,*” while recognizing the difficulty of balancing patient needs with team dynamics (PSW 17).

#### Being trusted and respected for doing this work (role valuing)

Trust emerged across participants’ accounts as a key condition shaping whether PSWs were supported to engage fully in prognostic conversations, influencing how their expertise was recognized, valued, and enacted within interprofessional teams.

#### Recognition of PSWs’ psychosocial clinical expertise

Across interviews, PSWs described feeling respected and valued for their clinical contributions to prognostic conversations, particularly within specialty palliative care teams. One PSW noted that, unlike other hospital teams that may expect social workers to focus on concrete needs, *“the palliative care team really values our clinical skill… they really see and appreciate our clinical skillset”* (PSW 17). Physicians similarly emphasized PSWs’ expertise, with one stating, *“.our social workers are more capable than most medical providers to be able to address prognosis and respond with emotion or demonstrate empathy and handle silence”* (MD 10).

#### Interprofessional respect and learning

Both professions highlighted mutual respect and interprofessional learning as central to effective collaboration. PSWs described their assessments of patient and family understanding as valued by physicians, with one noting, *“I know they value my assessment… and appreciate the education I provide about illness trajectory”* (PSW 16). Physicians described PSWs’ communication skills as enhancing team practice, emphasizing that social workers are *“very skilled and gifted in being present with patients*,*”* and noting that they *“often learn from how they ask questions or respond to emotion*,*”* which serves as a reminder to *“slow down and listen more carefully”* (MD 5a).

#### Trust as a condition for role enactment

Participants’ highlighted the centrality of relational conditions in influencing whether physicians’ supported PSWs engaging fully in prognostic conversations. Physicians expressed strong confidence in the judgment and communication skills of established team PSWs but described greater hesitancy with newer or non-specialist social workers. As one physician noted, *“we’ve all worked with our social workers for years and fully trust that they are going to appropriately take what we’re saying and talk to the patient… if there were someone new on the team*,* I would probably be a little bit more hesitant”* (MD 10). Thus, physicians’ trust and support for PSWs’ ability to work at the top of their license are influenced by their relationship and PSWs’ practice experience and skill set.

## Discussion

Findings suggest that cultivating prognostic awareness is best understood within these accounts as a specialty-level aspect of palliative social work practice, shaped by interprofessional relationships, team structures, and organizational context. By situating the findings within a role theory framework, we can see how role clarity, boundaries, sharing, and tensions shape PSWs’ ability to contribute to patients’ prognostic understanding. PSWs’ accounts highlight how advanced training, experience, and team trust shape their participation in extending prognostic discussions [5, 10, 44]. Role theory further underscores that these contributions are mediated by interprofessional trust, team expectations, and organizational conditions, suggesting that PSWs’ engagement in prognostic discussions reflects not only professional scope, but also the conditions that enable their expertise to be used effectively [45].

PSWs’ advanced practice in prognostic conversations is grounded in specialized training, accumulated clinical experience, and core professional frameworks such as psychodynamic, family systems, and person-in-environment perspectives [46, 47]. At the same time, findings reveal factors that may contribute to role strain, particularly in high-acuity hospital settings where workflow pressures, limited physician availability, or less-experienced clinicians may prompt PSWs to take on expanded responsibilities. PSW’s accounts suggest that these adaptive practices were not rooted in a desire to overstep professional boundaries, but rather reflected a commitment to patient care, team functioning, and professional ethics in the context of real-world practice.

Findings also suggest that PSWs’ professional roles may expand and contract in response to organizational context and team composition. In contexts where PSWs partnered with dedicated palliative physicians, role blurring was safe and valued. In contrast, when the PSW is the sole palliative care provider, primary team expectations lead them to adopt a broader scope that requires grounding in medical knowledge, advanced communication skills, and leadership skills to navigate inherent power dynamics within the medical system. These shifts reflect advanced practice skills such as situational awareness, flexibility, and boundary negotiation [44, 47]. They also point to important training implications, including the need for structured physician mentorship, advanced communication training, and facilitated reflection on boundary work as a core clinical competence.

Interprofessional trust and mutual respect emerged as central to effective role sharing. In well-integrated teams, role valuing and agreement created the conditions for collaborative practice, which may be particularly meaningful given that PSWs are often perceived by patients and families as less threatening. This relational positioning may support patient-centered communication while also enhancing team functioning. Consistent with prior work by Ma et al. [24], role expansion in serious-illness communication appears to benefit from organizational support, leadership endorsement, and cross-disciplinary agreement. Boundary work, as described by Chreim [13] and Farchi [48], suggests that well-defined boundaries can facilitate collaboration by making professional expertise visible rather than restrictive. By reinforcing interprofessional trust and clear boundaries, teams can maximize PSWs’ value in cultivating prognostic awareness, enabling them to practice at the full height of their expertise [13].

### Study limitations

This study reflects the perspectives of highly experienced, APHSW-certified palliative social workers, most of whom practiced in urban academic medical centers. As such, findings may not fully represent the experiences of generalist social workers, PSWs practicing in less-resourced settings, or teams without access to specialty palliative care physicians. Consistent with Interpretive Description, findings are not intended to be statistically generalizable but to offer practice-relevant insights that may be conceptually transferable to similar clinical contexts. Physician participants were identified by PSWs, which may have introduced selection bias toward clinicians with established collaborative relationships, and physician interviews were shorter than PSW interviews, potentially limiting depth in physicians’ accounts. The primary author’s professional background as a PSW also shaped the interpretation of the data; reflexive journaling and team dialogue were used to support transparency.

### Research and practice implications

Findings suggest that participants viewed cultivating prognostic awareness as a specialty-level intervention within the scope of PSW practice when done in collaboration with physicians. Clear role expectations, access to physician mentorship, and supportive team communication may help reduce role confusion and support PSWs in this work. Future research should examine how PSWs enact this role across diverse healthcare settings, included less resourced community hospitals as well as identify the specific clinical skills and decision-making processes that PSWs engage in to support patients’ prognostic understanding and to inform training and workforce development.

## Supplementary Information

Supplementary Material 1.

Supplementary Material 2.

Supplementary Material 3.

## Data Availability

The datasets generated and/or analyzed during the current study are not publicly available due to the confidential nature of the qualitative interview data but are available from the corresponding author on reasonable request.

## Abbreviations

APHSW: C–Advanced Palliative and Hospice Social Work Certification
IPC: Interprofessional Palliative Care
PSW: Palliative Social Worker
