# Supplementary material for: Top of the license practice or out of our scope? A qualitative analysis of social workers’ role in cultivating prognostic awareness on hospital palliative care teams
Source: BMC Palliat Care. 2026 Feb 25;25:78. doi: 10.1186/s12904-026-02028-w (PMC13041204; doi:10.1186/s12904-026-02028-w)
Supplement: Supplementary file 1 — Supplementary Material 1. [file 12904_2026_2028_MOESM1_ESM.docx]

Interview Guide: Interprofessional Team Members

Expected duration: 20-30 minutes

Hello. Thank you for taking the time to do this interview.

CONSENT

I emailed you a consent form on (date), were you able to read it? Do you have any questions?

In the consent form, I also asked if I could record this conversation, is that okay with you?

Great, I am going to read to you the final paragraph to confirm verbal consent: *I have read the information in this consent form, including risks and possible benefits. I have been given the chance to ask questions. My questions have been answered to my satisfaction, and I agree to participate in the study.*

Do you verbally consent to this? Thank you.

**DEMOGRAPHICS**

As part of the study, I hope to interview professionals working in palliative care from diverse backgrounds and experiences. Would you be willing to share a little information about your background?

1. Can you tell me when you started working as an identified Palliative care clinician?
2. How long have you been working on this Palliative Care team?
3. Would you be willing to share how you identify in terms of gender, race and/or ethnicity, and age?

**DEFINITION OF CULTIVATION OF PROGNOSTIC AWARENESS.**

As you know, my dissertation focuses on the “cultivation of prognostic awareness” by palliative care teams, with an emphasis on the role of social workers in this work. In the study, I define prognostic awareness as “a patient’s capacity to understand their prognosis and the likely trajectory of their illness.” Central to my work is a view that gaining an understanding of prognosis is a rather than a single event.

While giving a **time-based prognosis** is one way to provide information, I am taking a broader context or lens to understand how members of an interprofessional team work together, and individually, to cultivate patients’ and families’ understanding of their likely illness trajectory and prognosis. In essence, I want to explore the ways in which palliative care clinicians help move patients and families along the continuum of understanding.

1. As a starting point, I would be interested in hearing your initial thoughts or reactions to this definition of cultivation of prognostic awareness as a process and as involving the **entire team**. How does it align, or not align, with your own thoughts or views?

**2) CASE EXAMPLE**

Thank you, now I am going to present a short a patient scenario and then ask you about the team might handle it to gain some insights as to how your team operates.

Mark is a 63-year-old, married African American man who was diagnosed 5 months prior with Stage IV Pancreatic cancer with liver metastasis, he was treated with gemcitabine and radiation, after 6 cycles showed stable disease. Your team is consulted when he presents to the hospital reporting weight loss and abdominal pain with concerns around disease progression. He has not worked with your team before. The team is consulted for pain and goals of care. During the initial palliative care you and a member of your team meet with Mark and his family, during this time he states that the most important thing is walking his daughter down the aisle at her wedding in 6 months. He references a friend who has lived with cancer for many years and speaks about God’s will and his church community. All evidence suggests that while his desire to be at the wedding may be possible, you have concerns that he may not live long enough or be strong enough to fulfil his goal.

1. Following the meeting, what do you think the team’s goals and next steps would be?
2. How important do you feel that addressing his low prognostic awareness would be to the team? To you in your work?
3. Can you tell me what kind of strategy or skills the team might use in a scenario like this to address Mark’s statements around the wedding?
4. Is there anything you feel like you might do specifically in your role as the *(Insert profession here)?*
5. Can you tell me a bit about the team’s expectations of the role of the social worker in a case like this?

Thank you. Now I wanted to move a bit more specifically about your experience of working with a social worker as a part of the team.

Broadly, I am interested in the perceived role that palliative social workers play in the process of cultivating prognostic awareness. In part because there are a wide range of opinions about this – within social work and across other professions- Some people feel like is part of what a palliative social worker does and others feel it is outside of their scope. Would you be willing to share your initial thoughts about this?

3) In what ways, if any, do you feel a social worker contributes the cultivation of disease understanding as a part of their clinical work with patients and families?

4) Can you share with me any limitations or concerns you or others might have *if or when* social workers take this on as a part of their role?

5) Can you identify any other knowledge, skills or tricks you see the social worker bring to supporting patients and family in the cultivation of prognostic awareness?

6) Can you share with me a case or scenario where you feel social worker made a significant positive contribution to the cultivation of prognostic awareness?

Thank you so much for this interview, I really appreciate your time. Before we close, is there anything else you feel it is important for me to know about social workers’ roles in the cultivation of patients’ prognostic awareness? I haven’t asked or at any prior point that you would like to expand on or further explain?
