# Supplementary material for: Top of the license practice or out of our scope? A qualitative analysis of social workers’ role in cultivating prognostic awareness on hospital palliative care teams
Source: BMC Palliat Care. 2026 Feb 25;25:78. doi: 10.1186/s12904-026-02028-w (PMC13041204; doi:10.1186/s12904-026-02028-w)
Supplement: Supplementary file 2 — Supplementary Material 2. [file 12904_2026_2028_MOESM2_ESM.docx]

Interview Guide: Palliative Social Workers

Expected duration: 45-60 minutes

Hello! Thank you for taking the time to do this interview. As I shared in my email, the interview will take about 45-60 minutes. Is this still a good time to speak?

CONSENT

I emailed you a consent form on (date), were you able to read it? Do you have any questions?

In the consent form, I also asked if I could record this conversation, is that okay with you?

Great, I am going to read to you the final paragraph at the end of the form to confirm your verbal consent:

I have read the information in this consent form, including risks and possible benefits. I have been given the chance to ask questions. My questions have been answered to my satisfaction, and I agree to participate in the study. Do you verbally consent to this study? Thank you.

**DEMOGRAPHICS**

As part of the study, I hope to interview professionals working in palliative care from diverse backgrounds and experiences. Would you be willing to share a little information about your background?

1. Can you tell me when you got your MSW?
2. When did you start working in a Palliative care clinician position?
3. Would you be willing to share how you identify in terms of gender, race and/or ethnicity, and age?

**DEFINITION OF CULTIVATION OF PROGNOSTIC AWARENESS.**

As you know, my dissertation focuses on the “cultivation of prognostic awareness” by palliative care teams, with an emphasis on the role of social workers in this work.

In this study, I define prognostic awareness as “a patient’s capacity to understand their prognosis and the likely trajectory of their illness.” Central to my work is a view that gaining an understanding of prognosis is a **process that evolves over time** rather than a single event.

While giving a **time-based prognosis** is one way to provide information, I am taking a broader context or lens to understand how members of an interprofessional team work together, and individually, to cultivate patients’ and families’ understanding of their likely illness trajectory and prognosis. In essence, I want to explore the ways in which palliative care clinicians help move patients and families along the continuum of understanding.

1. As a starting point, I would be interested in hearing your initial thoughts or reactions to this definition of cultivation of prognostic awareness as a ***process*** and as involving the **entire team**. How does it align, or not align, with your own thoughts or views?

**2) CASE EXAMPLE**

Thank you, now I am going to present a short patient scenario and then ask you about how your team might handle it to gain some insights as to how your team operates.

Mark is a 63-year-old, married African American man who was diagnosed 5 months prior with Stage IV Pancreatic cancer with liver metastasis, he was treated with gemcitabine and radiation, after 6 cycles showed stable disease. Your team is consulted when he presents to the hospital reporting weight loss and abdominal pain with concerns around disease progression. He has not worked with your team before. The team is consulted for pain and goals of care. During the initial palliative care you and a member of your team meet with Mark and his family, during this time he states that the most important thing is walking his daughter down the aisle at her wedding in 6 months. He references a friend who has lived with cancer for many years and speaks about God’s will and his church community. All evidence suggests that while his desire to be at the wedding may be possible, you have concerns that he may not live long enough or be strong enough to fulfil his goal.

1. Following the meeting, what do you think the team’s goals and next steps would be?
2. How important do you feel that addressing his low prognostic awareness would be to the team? To you in your work?
3. Can you tell me what kind of strategy or skills the team might use in a scenario like this to address Marks statements around the wedding?
4. Is there anything you feel like you might do specifically in your role as the social worker?
5. What are the team’s expectation of the role of the social worker in a case like this?

3) Do you feel like your team generally supports or discourage your engagement in cultivating prognostic awareness as a part of your social work practice? What about your Social work Department? And the hospital?

4) Can you comment on how your palliative team is similar or different to other interprofessional teams you work with, in terms of your role and their expectations of you in case situations like this?

5) Do you think the role you play on the palliative care team is typical or atypical of what a other hospital-based social workers experience on teams?

**IN-DEPTH DISCUSSION OF SOCIAL WORKERS’ INTEGRATION OF PROGNOSTIC AWARENESS IN WORK-OVERVIEW**

Thank you, so now I am going to focus im more deeply on your role as a social worker.

I am interested in the perceived role that palliative social workers play in the process of cultivating prognostic awareness. In part because there are a wide range of opinions about this – within social work and across other professions- Some people feel like is part of what a palliative social worker does and others feel it is outside of their scope. I would like to get your insights and perspectives regarding this.

6) Would you define the cultivation of prognostic awareness as a part of your clinical practice? Why or why not?

1. Looking at it a bit more broadly, can you explain why you believe it was important to integrate information on the illness trajectory or prognosis into your work/conversations with the patient or family?
2. Can you share the 3 or 4 most common clinical tasks or interventions in which a patient’s prognostic awareness comes up in your work with patients and families? Ie HCP

9) Can you give examples of the types of situations in which you are typically comfortable or confident discussing illness trajectory or prognosis with patients and families?

10) Can you give examples of types of situations in which you feel less comfortable, less confident, and/or avoid discussing illness trajectory or prognosis with patients?

Thanks, we are getting to the end of this interview and I want to dig in a bit deeper around the the actual ways or skills social workers use when they are discussing prognostic or disease trajectory with patients.

11) Can you share with me any knowledge, skills, strategies, or maybe “tricks of the trade” you use that you have found helpful when supporting patients and family along this increased understanding?

12) How do you feel you developed your skills in cultivating prognostic awareness? Was it formal training or on-the-job experience?

Thank you so much for this whole interview. I really appreciate your time. Before we close, is there anything else you feel it is important for me to know about social workers’ roles in the cultivation of patients prognostic awareness I haven’t asked or a prior point that you would like to expand on or further explain?
