# Supplementary material for: Top of the license practice or out of our scope? A qualitative analysis of social workers’ role in cultivating prognostic awareness on hospital palliative care teams
Source: BMC Palliat Care. 2026 Feb 25;25:78. doi: 10.1186/s12904-026-02028-w (PMC13041204; doi:10.1186/s12904-026-02028-w)
Supplement: Supplementary file 3 — Supplementary Material 3. [file 12904_2026_2028_MOESM3_ESM.docx]

**Supplementary Material: Full Quotes by category.**

**Table 1: Negotiating PSWs’ role in prognostic awareness (Role Agreement)**

| **Subtheme** | **Speaker** | **Illustrative Quote** |
| --- | --- | --- |
| Cultivating PA is within PSW Scope of practice | PSW 1 | Part of our job is to help folks define their goals of care...prognostic awareness is intimately tied to those…you can't tease it out. |
|  | MD 10 | It's an essential part of what we do as an palliative care team... understanding the timeline is important in decision making...it takes everyone, especially the social worker, to help patients and families make sense of what’s ahead. |
| Cultivating PA is Specialist-level skill | PSW 2 | Cultivating prognostic awareness is an advanced palliative care skill. I wouldn’t expect somebody [early in their career] to feel as comfortable  as I am after 10 years. |
|  | MD 16 | **Palliative care has** begun to think of prognostication more broadly **than what I learned in medicine, moving from a concrete, ‘We must help them understand how much time they have left’ to ‘Let's work as a team to help paint a picture for what the future could look like, continue to revise it, cope as best we can, and move it along’… which is different and takes a high level of skill.** |

### **Table 2: Drawing boundaries around prognostic work (Role boundaries)**

| Subtheme | Speaker | Illustrative Quote |
| --- | --- | --- |
| Time-based prognoses | PSW 2 | I'm not coming up with a prognosis on my own; that is out of our scope. ...but if the doctor is telling me the prognosis-- It is 100% within my scope to take that ... and translate that into my work with a family. |
| Medically complex discussions | MD 4 | I’d be concerned if a social worker gave a strong opinion on a medical procedure or talked about time without physician input… it’s important that treatment decisions come from the physician. |
| Primary team consultations | PSW 15 | There are times when the primary team asks us not to get into detailed prognosis... we have to decide if we will respect their request, or are we doing a disservice to the patient if we withhold too much. |

**Table 3: Shared and collaborative prognostic work across professions (Role sharing)**

| Subtheme | Speaker | Illustrative Quote |
| --- | --- | --- |
| Joint consults | PSW 2 | Joint consults are like a dance... It's collaborative. Sometimes I ask a doctor-type question, and they ask one more psychosocial. |
|  | MD 17 | It's a team sport, and if one individual delivers all of the information it can become an emotional avalanche… social workers help by reflecting back things the medical provider might have missed because they’re focused on the next phase of the conversation. |
| Extending conversations | PSW 8 | I often circle back later… helping them process and move through difficult emotions so they can hear and understand the prognostic information the team is trying to deliver. |
|  | MD 5a | I hear my social worker on the phone with patients and families having deeper and more difficult conversations than we even have in our meetings. That’s really helpful, because by the time we come together for a family meeting, she’s already helped them process a lot of what’s happening. It makes those conversations more productive and compassionate. |
| Centering lived experience | PSW 15 | I explore their lived experience, then give a functional prognosis... 'You've been in the hospital 3 times in the last 4 months, I'm worried this is the best it's going to get.' It’s saying time is getting shorter, but not directly. |
| PSW’s are seen differently than MDs | MD 3a | Social workers talk about things like functional decline or what support might be needed… It's seen differently than when I talk about it... somehow it feels less threatening. |

**Table 4: Navigating tensions and competing expectations in practice (Role tension and potential role conflict)**

| Subtheme | Speaker | Illustrative Quote |
| --- | --- | --- |
| Primary team  expectations | PSW 2 | There have been multiple times when one of our palliative care providers are not available, I am asked to step in…even if the conversation we are having is changing code status…I think the role expectations are a little bit different from that standpoint. |
|  | MD 15 | **Our team social workers are often consulted alone to have code status discussions. I often feel bad, it’s, realyl that’s not their role as a social worker, but I guess they are seen as “palliative care,” so maybe it is expected?** |
| Workflow Pressures | PSW 9 | If the team is swamped… I can lay the groundwork...then we can circle back later together to have a deeper conversation. |
|  | MD 11 | More often than not, we’re being pulled in too many directions. … If I can’t be there for the family meeting, she’ll (PSW) often go in my place as the sole palliative provider”. |
| Working with less  experienced clinicians | MD 16 | Our palliative social worker will sometimes step in and say, ‘Maybe it would be helpful if you talk a little about how this is going to look in the future,’ just to guide the discussion back toward prognosis or planning. It’s done so gently that it doesn’t feel intrusive, and it’s really effective—particularly with less experienced providers who might otherwise skip over that part.” |
|  | PSW 13 | Sometimes the doctors are just not good communicators... there are times when I'm with newer doctors, I will coach them 'Say the word death,' or I'll even write it and slide it to the doctor... but sometimes you just have to say it directly. |
| Ethical obligations | PSW 11 | I had to say something... the doctor wasn't going to, and it was unethical to let her keep asking without an answer. |
|  | PSW 17 | We had a BMT patient who we all knew was dying, but no one had the courage to say it. PSWs get really worried about those kinds of situations and feel ethically obligated…that often drives me to say what no one else is saying. |

**Table 5: Being trusted and respected for doing this work (role valuing)**

| **Subtheme** | **Speaker** | **Illustrative Quote** |
| --- | --- | --- |
| Recognition of clinical skills | MD 10 | Honestly, our social workers are more capable than most medical providers to be able to address prognosis and respond with emotion or demonstrate empathy and handle silence... |
|  | PSW 17 | The palliative care team really values our clinical skill more than other teams who may expect we only address concrete needs, transportation vouchers and things of that sort. **They really see and appreciate our clinical skillset.** |
| Team respect | PSW 16 | I know they value my assessment of patient and family understanding of their diagnosis and appreciate the education I provide about illness trajectory. |
|  | MD 5a | The social workers on my team are very skilled and gifted in being present with patients. They have a way of sitting in silence that I really admire. They’re trained in communication in ways that I’m not. I often learn from how they ask questions or respond to emotion. It reminds me to slow down and listen more carefully. |
| Mutual Trust | MD 5b | We've worked with our PSWs for years and fully trust that they will appropriately take what we're saying and talk to patients... but if it was a PSW I didn't know, I would be more hesitant." |
|  | MD 15 | It totally depends on whether or not you know the person. … If I don’t know the person it’s really hard to assess that, because I’m like, ‘I don’t know you and don’t know if I can I trust you.’ |
